# Clinical outcomes and a nomogram for de novo metastatic breast cancer with lung metastasis: a population-based study

**DOI:** 10.1038/s41598-022-07565-x

**Published:** 2022-03-04

**Authors:** Weiming Liu, Yiqun Han

**Affiliations:** 1 China Rehabilitation Research Center, Beijing Boai hospital, Beijing, China; 2 National Cancer Center/National Clinical Research Center for Cancer/Cancer Hospital, Chinese Academy of Medical Sciences and Peking Union Medical College, Beijing, China

**Keywords:** Cancer, Biomarkers, Medical research, Oncology, Risk factors

## Abstract

To better understand the clinical characteristics of newly diagnosed lung metastatic breast cancer (LMBC) and quantify its prognosis, we retrieved data on patients with LMBC from the Surveillance, Epidemiology, and End Results database. Eligible patients were randomly assigned to training and validation cohorts (ratio 7:3) to establish a nomogram using the Cox proportional hazards regression model. In total, 4310 patients with LMBC were enrolled, including 52.4% (2259/4310) HR+/HER2−, 17.6% (757/4310) HR+/HER2+, 10.8% (467/4310) HR−/HER2+, and 19.2% (827/4310) HR−/HER2− subtype patients. Inclinations of lung and brain involvement in HR−/HER2+ and HR−/HER2− subgroups, liver involvement in the HER2 overexpressing subgroup, and bone involvement in the HR-positive subgroup were detected in the LMBC population. Regarding prognosis, HR+/HER2+ subtype patients presented the most favorable profile (mOS 35.0 months, 95% CI 30.1–39.9), while HR−/HER2− patients exhibited the worst (mOS 11.0 months, 95% CI, 10.0–11.9). A nomogram was developed in the training cohort and validated internally (C-index 0.70) and externally (C-index 0.71), suggestive of decent performance. This study assessed the clinical outcomes associated with molecular subtypes, metastatic patterns, and surgical intervention and provided a robust nomogram for the estimation of survival probabilities, which are promising for the management of LMBC in clinical practice.

## Introduction

De novo metastatic breast cancer refers to distant metastasis at the initial diagnosis and an inferior prognosis, with a 5-year survival rate of less than 30%; patients with de novo metastatic breast cancer account for approximately 5% of the entire population^1,2^. Although it is treatable considering the advances in novel therapeutics, de novo metastatic disease tends to be incurable and could be a therapeutic challenge in clinical practice. Among this group of diseases, the occurrence of lung metastasis is estimated to be 21–77%; the lung is one of the most common sites of cancer spread^3–5^.

Despite the notable prevalence of lung metastatic breast cancer (LMBC), limited studies have evaluated the presentations of patients with LMBC. Hence, the clinicopathological features and prognostic profiles are unclear. Previous studies have reported preliminary findings regarding the molecular subtypes and lung metastasis^6^. However, this association needs to be adequately studied due to insufficient clinical outcomes and follow-up. In addition, the tumor burden at initial diagnosis could be a critical factor, and the metastatic pattern is a significant component for cancer management and survival prediction of de novo disease. However, few studies have focused on this profile in the LMBC population. Moreover, as a predominant treatment, surgical intervention could be the foremost option for early breast cancer, but its prognostic benefits have not been adequately determined for de novo metastatic disease^7–9^. Therefore, the prognostic value of surgical performance in the therapeutic course of patients with LMBC should be clarified.

We conducted this study to comprehensively discuss the clinicopathological and prognostic characteristics of patients with LMBC to assess the associations between clinical outcomes and molecular subtypes, metastatic patterns, and surgical performance. We further aimed to establish a prediction model for the individual estimation of survival probabilities of patients with LMBC to provide promising evidence and reference for the introduction of individual therapeutics for patients with LMBC in clinical practice.

## Methods

### Population

Data on patients diagnosed with breast cancer between January 1, 2010 and December 31, 2016 were obtained from the Surveillance, Epidemiology, and End Results (SEER) database. Patients who were newly diagnosed with LMBC and had no missing clinicopathological and survival data were assessed for eligibility. Patients were excluded if (1) tumor grade; molecular subtypes; and the status of estrogen receptor (ER), progesterone receptor (PgR), and human epidermal growth factor receptor 2 (HER2), in addition to that of visceral metastases, were unknown and (2) tumor size and node involvement were not evaluated. Data analyses were performed in December 2020.

Information on the selected cohort was successively extracted for the analysis of the following: age at diagnosis, sex, race, laterality, histologic type, grade, molecular subtypes, immunochemical status (ER, PR, and HER2), tumor size, node involvement, visceral metastases, performance of surgery, radiotherapy, and chemotherapy. This study was conducted in accordance with the Strengthening the Reporting of Observational Studies in Epidemiology guidelines^10^ and the Transparent Reporting of a Multivariate Prediction Model for Individual Prognosis or Diagnosis statement^11^.

### Outcome

LMBC was defined as de novo metastatic breast cancer presenting with lung metastasis with positive histological confirmation. The differences in clinicopathological features and prognosis were compared among the molecular subtypes, which were classified into four categories—hormone receptor (HR)-positive/HER2-negative (HR+/HER2−), HR-positive/HER2-positive (HR+/HER2 +), HR-negative/HER2-positive (HR−/HER2+, HER2), and HR-negative/HER2-negative (HR−/HER2−, TN). Overall survival (OS) was defined as the interval between the initial diagnosis of breast cancer and death caused by any reason. According to SEER terminology, visceral metastases involve the liver and brain. The American Joint Committee on Cancer 7th edition guidelines were adopted to define the tumor–node–metastasis stage of breast cancer.

### Statistical analysis

Comparative analysis of baseline characteristics was performed using Pearson’s chi-square test and Fisher’s exact probability test for qualitative data and the t-test or Wilcoxon rank test for quantitative data with a normal and abnormal distribution, respectively. Survival outcomes were compared using the Kaplan–Meier method with log-rank tests. Patients were randomly assigned to the training and validation cohorts in a 7:3 ratio to establish and externally validate the model. Prognostic factors were identified with consecutive performance of univariate and multivariate Cox proportional hazards regression analyses, which were adopted to develop a nomogram for estimating the 2- and 5-year survival probabilities. The discriminative and calibrating capabilities of this nomogram were evaluated both internally and externally using the concordance index (C-index) and calibration curves with bias-corrected validation under 1000 bootstrap resamples. A C-index of 0.5 indicated agreement by chance, and a C-index of 1 indicated perfect discrimination. All statistical analyses were two sided, with *P* < 0.05 considered statistically significant, and were performed using IBM SPSS Statistics (version 26.0; IBM Corp., Armonk, NY), and R software (version 3.6.4, www.r-project.org/).

## Results

Among the 7746 initially identified patients with LMBC, 4310 were finally eligible (Supplementary Fig. 1). The population demographics and baseline clinicopathological characteristics are presented in Supplementary Table 1.

### Clinical outcomes associated with molecular subtypes

In total, 52.4% (2259/4310) patients were HR+/HER2−, 17.6% patients (757/4310) were HR+/HER2+, 10.8% (467/4310) patients were HR−/HER2+, and 19.2% (827/4310) patients were HR−/HER2−. Their baseline features are listed in Table 1. The median age at diagnosis in patients with patients HR+/HER2−, HR+/HER2+, HR−/HER2+, and HR−/HER2− subtypes was 64.0, 59.0, 59.0, and 62.0 years, respectively, and there was profound heterogeneity in the disease characteristics among them. Compared to luminal-like subtype disease, the HER2 and TN subtypes of LMBC presented a higher grade (*P* < 0.0001), a larger tumor size (*P* < 0.0001), a higher rate of node involvement (*P* < 0.0001), and a higher incidence of brain metastasis (*P* < 0.0001). Luminal-like subtype LMBC exhibited a higher rate of bone metastasis (*P* < 0.0001), while the HER2 overexpression subtype, including HR+/HER2+ and HR−/HER2+, tended to be associated with a relatively higher occurrence of liver metastasis (*P* < 0.0001).

**Table 1:** Population demographics and baseline characteristics of included patients associated with molecular subtypes.

Characteristics	HR+/HER2− (N = 2259)		HR+/HER2+ (N = 757)		HR−/HER2+ (N = 467)		HR−/HER2− (N = 827)		*P* value
	No	Percent (%)	No	Percent (%)	No	Percent (%)	No	Percent (%)	
Age, years	64.0		59.0		59.0		62.0		< 0.0001
**Age group, years**									< 0.0001
< 50	345	15.3	147	19.4	103	22.1	164	19.8	
50–69	1141	50.5	430	56.8	236	50.5	426	51.5	
≥ 70	773	34.2	180	23.8	128	27.4	237	28.7	
**Sex**									0.013
Female	2217	98.1	742	98.0	466	99.8	820	99.2	
Male	42	1.9	15	2.0	1	0.2	7	0.8	
**Race**									< 0.0001
White	1662	73.6	548	72.4	331	70.9	555	67.1	
Black	385	17.0	130	17.2	87	18.6	215	26.0	
Others	212	9.4	79	10.4	49	10.5	57	6.9	
**Laterality**									0.986
Left	1133	50.2	388	51.3	240	51.4	413	49.9	
Right	1115	49.4	364	48.1	225	48.2	410	49.6	
Others	11	0.5	5	0.7	2	0.4	4	0.5	
**Histologic type**									< 0.0001
DC	1826	80.8	658	86.9	405	86.7	676	81.7	
LC	118	5.2	11	1.5	4	0.9	5	0.6	
Others	315	13.9	88	11.6	58	12.4	146	17.7	
**Grade**									< 0.0001
Grade1	211	9.3	21	2.8	1	0.2	8	1.0	
Grade2	1135	50.2	271	35.8	96	20.6	125	15.1	
Grade3	898	39.8	461	60.9	362	77.5	682	82.5	
Grade4	15	0.7	4	0.5	8	1.7	12	1.5	
**T**									< 0.0001
T0	6	0.3	0	0.0	1	0.2	5	0.6	
T1	274	12.1	79	10.4	40	8.6	70	8.5	
T2	669	29.6	223	29.5	115	24.6	217	26.2	
T3	364	16.1	130	17.2	86	18.4	181	21.9	
T4	946	41.9	325	42.9	225	48.2	354	42.8	
**N**									< 0.0001
N0/N1mi	569	25.2	135	17.8	104	22.3	225	27.2	
N1	1084	48.0	393	51.9	217	46.5	368	44.5	
N2	291	12.9	113	14.9	70	15.0	82	9.9	
N3	315	13.9	116	15.3	76	16.3	152	18.4	
**Bone involvement**									< 0.0001
Yes	1398	61.9	420	55.5	185	39.6	277	33.5	
No	861	38.1	337	44.5	282	60.4	550	66.5	
**Liver involvement**									< 0.0001
Yes	507	22.4	269	35.5	178	38.1	228	27.6	
No	1752	77.6	488	64.5	289	61.9	599	72.4	
**Brain involvement**									< 0.0001
Yes	173	7.7	82	10.8	58	12.4	109	13.2	
No	2086	92.3	675	89.2	409	87.6	718	86.8	
**Surgery**									< 0.0001
Yes	628	27.8	232	30.6	172	36.8	339	41.0	
No/unknown	1631	72.2	525	69.4	295	63.2	488	59.0	
**Radiotherapy**									0.750
Yes	653	28.9	206	27.2	131	28.1	244	29.5	
No/unknown	1606	71.1	551	72.8	336	71.9	583	70.5	
**Chemotherapy**									< 0.0001
Yes	1077	47.7	579	76.5	363	77.7	600	72.6	
No/unknown	1182	52.3	178	23.5	104	22.3	227	27.4	

**Figure 1:**
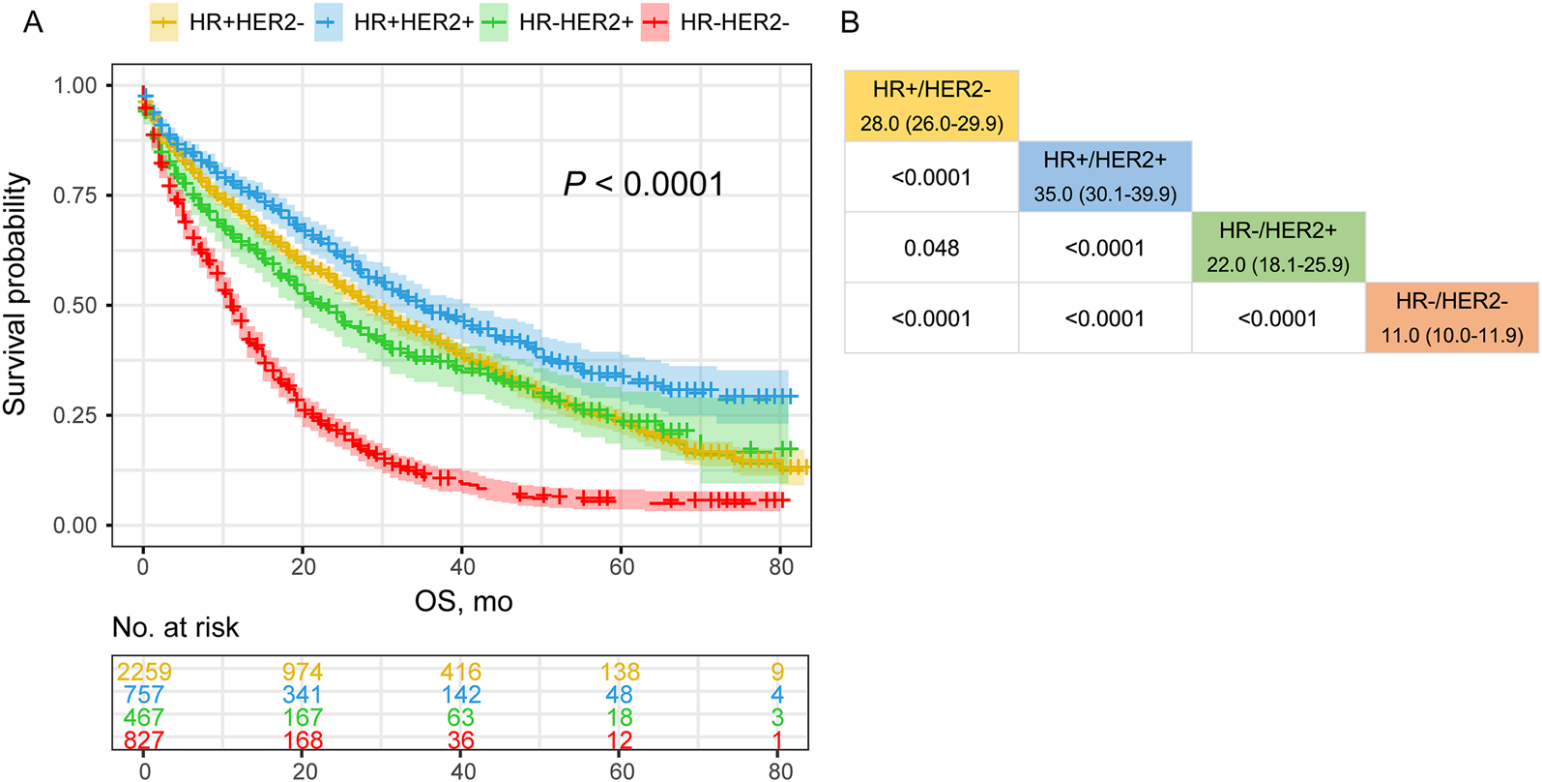
Comparative analysis of OS associated with molecular subtypes. (R software version 3.6.4, www.r-project.org).

Regarding prognosis related to molecular subtypes, the median OS was 35.0 months (95% confidence interval [CI] 30.1–39.9) in HR+/HER2+, 28.0 months (95% CI 26.0–29.9) in HR+/HER2−, 22.0 months (95% CI 18.1–25.9) in HR−/HER2+ and 11.0 months (95% CI 10.0–11.9) in HR−/HER2− subtypes, indicating a successively worse trend in overall prognosis (*P* < 0.0001; Fig. 1).

### Clinical outcomes associated with metastatic patterns

The metastatic patterns of patients with LMBC were analyzed; the involved cases and their survival were analyzed for outcome evaluation. Overall, lung-only metastatic disease had the highest incidence rate (1555/4310, 36.1%), followed by lung and bone metastatic disease (1332/4310, 30.9%), with no statistical significance in the median OS between the groups (*P* = 0.053; Supplementary Tables 2, 3). With respect to the number of metastatic sites, the overall prognosis constantly worsened with an increase in the number of involved organs (Supplementary Fig. 2A). For patients with malignancy involving three sites, an inferior tendency was detected in patients with bone, lung, and brain metastases (*P* < 0.0001; Supplementary Fig. 2B). However, no statistical significance was noted in the prognosis of patients with malignancy involving three sites (Supplementary Fig. 2C). In addition, patients with LMBC and brain metastasis exhibited the worst survival, and the additional involvement of the bone tended to exert little effect on the prognosis of patients with lung-only (*P* = 0.053); lung and liver (*P* = 0.621); and lung, liver, and brain metastasis (*P* = 0.648; Supplementary Table 3, Supplementary Fig. 2D).

### Clinical outcomes associated with treatment

The prognostic benefits of surgical performance were assessed in patients with de novo LMBC. Regarding molecular subtypes, a constantly improved OS was revealed across HR+/HER2+, HR+/HER2−, HR−/HER2+, and HR−/HER2− subtype disease (Supplementary Fig. 3A–D), which was consistent with the prognostic outcomes of patients with lung-only and paired-organ metastases with bone, liver, and brain involvement (Supplementary Fig. 4A–D). For the entire LMBC population, the overall OS was significantly improved by surgical intervention (*P* < 0.0001), and the comparative prognosis stratified by clinical characteristics is presented in Supplementary Table 4.

In addition, treatment patterns were subjected to comparative analysis in terms of survival benefits. A comparable effectiveness was detected between surgery plus chemotherapy (40.9 months, 95% CI 43.9–38.0) and surgery plus radiotherapy (42.0 months, 95% CI 48.8–35.2). In addition, no additional benefit was retrieved from surgery plus chemotherapy plus radiotherapy. The surgery-based combination regimen was advantageous compared to the other treatment options, including surgery alone, chemotherapy alone, or chemotherapy plus radiotherapy.

### Development and validation of the nomogram

Eligible patients were randomly allocated to the training and validation cohorts, which included 3017 and 1293 individuals, respectively. In the training cohort, the prognostic factors were successively identified, including age at diagnosis (*P* < 0.0001), race (*P* < 0.0001), histologic type (*P* = 0.001), tumor grade (*P* < 0.0001), molecular subtype (*P* < 0.0001), AJCC T stage (*P* = 0.006), bone metastasis (*P* < 0.0001), liver metastasis (*P* < 0.0001), brain metastasis (*P* < 0.0001), performance of surgery (*P* < 0.0001), and chemotherapy (*P* < 0.0001), which were collectively adopted to develop the prognostic model (Table 2). The nomogram showed that a tumor grade, molecular subtype, and age at diagnosis had a higher effect. The points of each variable were summed up by locating the respective points on the scale and then a straight line was drawn down to the total point scales to estimate the 2-year and 5-year survival rates.

**Table2:** Prognostic factors identified by uni- and multivariate COX regression analyses in the training cohort.

Characteristics	Univariate		Multivariate	
	HR (95%CI)	*P* value	HR (95%CI)	*P* value
**Age group, years**		< 0.0001		< 0.0001
< 50	Reference		Reference	
50–69	1.10 (0.96–1.26)	0.180	1.16 (1.01–1.33)	0.032
≥ 70	1.55 (1.35–1.79)	< 0.0001	1.61 (1.38–1.87)	< 0.0001
**Sex**		0.135		–
Female	Reference		–	–
Male	0.74 (0.51–1.10)	0.135	–	–
**Race**		< 0.0001		< 0.0001
White	Reference		Reference	
Black	1.29 (1.15–1.45)	< 0.0001	1.35 (1.20–1.52)	< 0.0001
Others	0.79 (0.66–0.96)	0.016	0.85 (0.70–1.02)	0.080
**Laterality**		0.844		–
Left	Reference		–	–
Right	0.97 (0.89–1.07)	0.599	–	–
Others	1.08 (0.54–2.17)	0.830	–	–
**Histologic type**		0.034		0.001
DC	Reference		Reference	
LC	1.23 (0.97–1.57)	0.086	1.41 (1.10–1.81)	0.006
Others	1.15 (1.01–1.32)	0.037	1.21 (1.05–1.38)	0.007
**Grade**		< 0.0001		< 0.0001
Grade1	Reference		Reference	
Grade2	1.28 (1.01–1.63)	0.043	1.34 (1.05–1.71)	0.018
Grade3	1.80 (1.42–2.28)	< 0.0001	1.87 (1.46–2.40)	< 0.0001
Grade4	2.07 (1.19–3.61)	0.010	2.06 (1.17–3.63)	0.012
**Subtype**		< 0.0001		< 0.0001
HR+/HER2−	Reference		Reference	
HR+/HER2+	0.81 (0.70–0.94)	0.005	0.92 (0.79–1.07)	0.267
HER2	1.15 (0.97–1.35)	0.108	1.37 (1.14–2.64)	0.001
TN	2.36 (2.10–2.65)	< 0.0001	2.77 (2.42–3.18)	< 0.0001
**T**		< 0.0001		0.006
T0	Reference		Reference	
T1	0.69 (0.29–1.69)	0.420	0.77 (0.32–1.89)	0.573
T2	0.72 (0.29–1.74)	0.462	0.76 (0.32–1.85)	0.551
T3	0.79 (0.33–1.92)	0.606	0.81 (0.33–1.96)	0.639
T4	0.91 (0.38–2.19)	0.828	0.94 (0.39–2.26)	0.883
**N**		0.106		–
N0/N1mi	Reference		–	–
N1	0.98 (0.87–1.10)	0.727	–	–
N2	0.83 (0.70–0.98)	0.026	–	–
N3	0.92 (0.79–1.07)	0.264	–	–
**Bone involvement**		< 0.0001		< 0.0001
Yes	Reference		Reference	
No	0.84 (0.76–0.92)	< 0.0001	0.79 (0.71–0.88)	< 0.0001
**Liver involvement**		< 0.0001		< 0.0001
Yes	Reference		Reference	
No	0.56 (0.51–0.62)	< 0.0001	0.62 (0.54–0.73)	< 0.0001
**Brain involvement**		< 0.0001		< 0.0001
Yes	Reference		Reference	
No	0.58 (0.50–0.67)	< 0.0001	0.56 (0.51–0.63)	< 0.0001
**Surgery**		< 0.0001		< 0.0001
No/unknown	Reference		Reference	
Yes	0.65 (0.59–0.73)	< 0.0001	0.67 (0.60–0.75)	< 0.0001
**Radiotherapy**		0.757		–
No/unknown	Reference		–	–
Yes	0.98 (0.89–1.09)	0.757	–	–
**Chemotherapy**		< 0.0001		< 0.0001
No/unknown	Reference		Reference	
Yes	0.69 (0.63–0.76)	< 0.0001	0.56 (0.49–0.62)	< 0.0001

**Figure 2:**
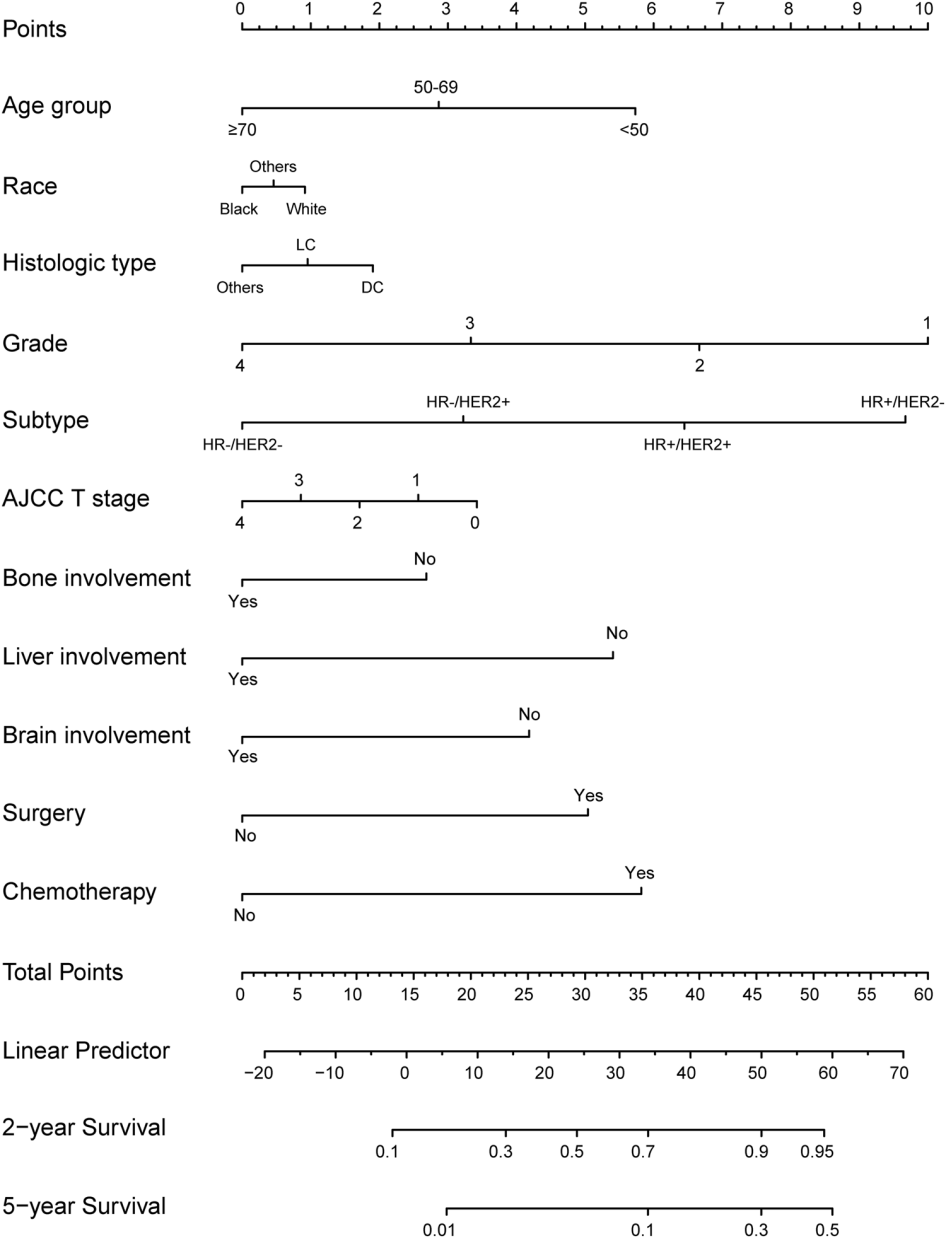
Nomogram for individual estimation of 2- and 5-year survival probabilities in LMBC patients. (R software version 3.6.4, www.r-project.org).

**Figure 3:**
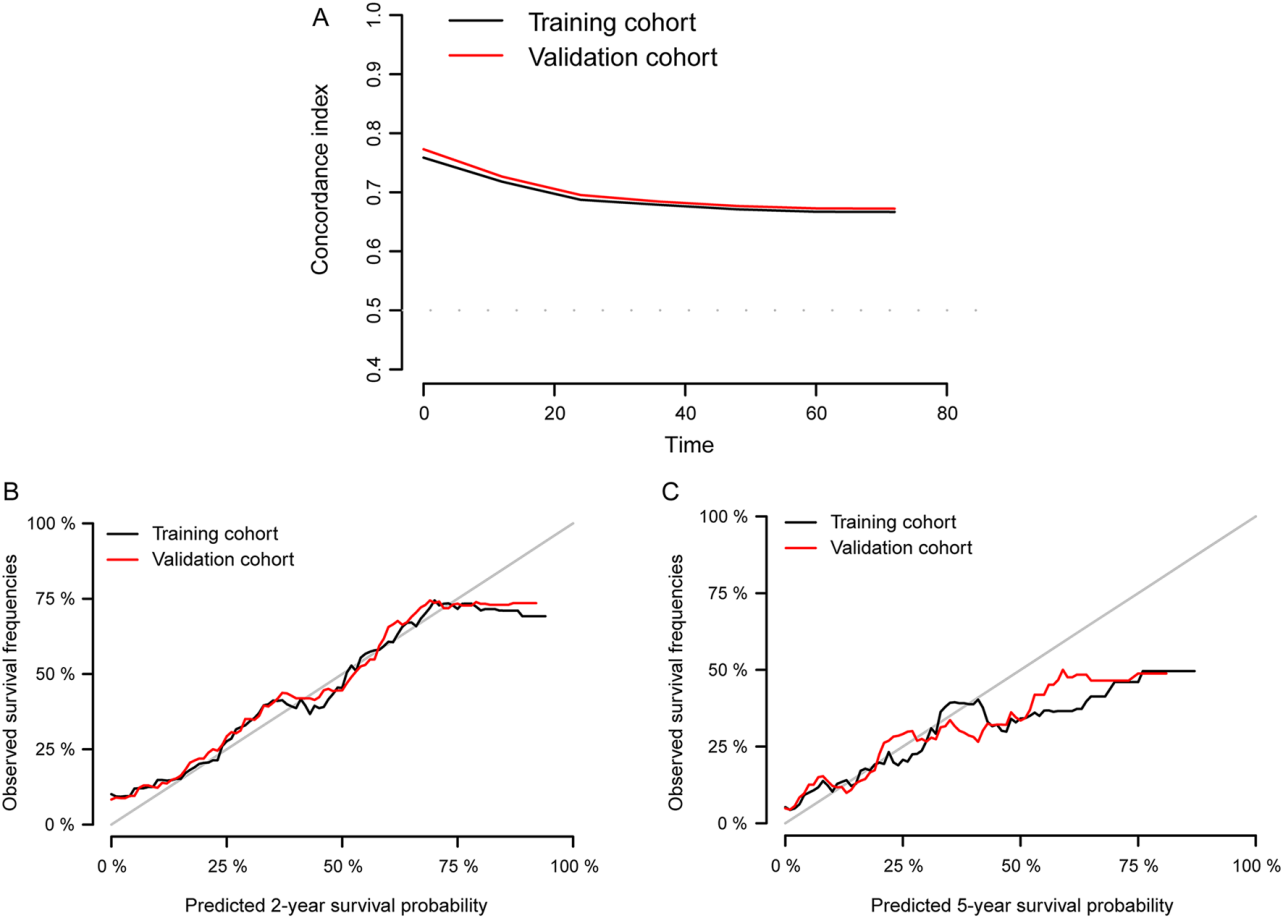
Validation of nomogram in the training cohort and validation cohort. **(A)** The C-index curves of nomogram in both the training and validation cohorts. **(B)** Calibration curves of 2-year survival rates in the training and validation cohorts. **(C)** Calibration curves of 5-year survival rates in the training and validation cohorts. (R software version 3.6.4, www.r-project.org).

The nomogram constructed for the estimation of 2- and 5-year survival in patients with LMBC was constructed is shown in Fig. 2. The overall C-index was 0.70 (95% CI 0.69–0.83) in the training cohort and 0.71 (95% CI 0.68–0.72) in the validation cohort, and the time-dependent C-index curves of the two cohorts signified that the values associated with survival were consistently > 0.50, indicative of favorable discriminative power (Fig. 3A). Calibration plots of the two cohorts demonstrated a decent agreement between the actual and predicted 2- and 5-year survival probabilities, which suggested a satisfactory calibration capability (Fig. 3B,C). In summary, the newly established nomogram showed good performance for survival estimation in patients with LMBC.

## Discussion

To our knowledge, this is the first study to comprehensively discuss the clinical features and prognostic outcomes associated with molecular subtypes, metastatic patterns, and surgical intervention and to develop a robust prediction model for the estimation of individual prognosis of de novo metastatic breast cancer with lung involvement.

To illustrate the distinctive presentations associated with molecular subtypes, we first performed comparative analyses among the LMBC population with HR+/HER2+, HR+/HER2−, HR−/HER2+ and HR−/HER2− subtype disease. The percentage of TN and HER2 subtype disease was relatively higher in patients with LMBC than in the entire breast cancer population (approximately 10% vs. 4%)^2^, suggesting an inclination of lung metastasis related to molecular subtype in patients with LMBC. An ascending tendency of lung involvement in TN and HER2 subtype breast cancer was noted in previous studies, with a recorded incidence of 20.8–35.0% and 22.9–45.0%, respectively^12–14^. In addition, we demonstrated that bone involvement tended to occur in luminal-like disease, while liver metastasis tended to occur in HER2 overexpression disease, which is consistent with the findings of previous studies that focused on de novo metastatic breast cancer^12,15,16^. The current evidence suggests that this kind of presentation can be independent of disease characteristics^17^, and our study demonstrated that the organ-specific metastasis remained stable in patients with initial lung metastasis. This type of subtype-associated predisposition could potentially constitute the intrinsic profiles of breast malignancies and provide clinical implications for organic selectivity in the management of cancer metastasis.

We also assessed the heterogeneous prognosis among the different molecular subtypes of LMBC, and our results suggested that the survival was in great favor of the HR+/HER2+ subtype, and patients with TN exhibited a relatively worse prognosis than the other subtypes. It is well acknowledged that TN breast cancer presents the most unfavorable disease features, with a median OS of 10–13 months in de novo metastatic breast cancer^18,19^, which was in line with the survival outcomes reported in the present study. In contrast, patients with HR+/HER2+LMBC had relatively favorable prognostic profiles, which could be the result of multiple treatment options for this type of subtype, including anti-HER2-targeted therapy and endocrine therapy. However, we could not further discuss the therapeutic influences on prognosis due to insufficient information on treatment in SEER database.

This is the first study to show that the distinctive survival outcomes are associated with metastatic profiles. We classified the metastatic patterns and further investigated the effects of the involved sites on the prognosis of LMBC. The prognosis gradually worsened as the total number of involved sites increased, and for patients with LMBC with paired metastatic sites, a successively inferior tendency was detected in lung involvement combined with bone, liver, and brain involvement. However, no statistical significance was revealed in patients with LMBC and three concurrent metastases. To further clarify the prognosis of patients with LMBC with diverse metastatic patterns, we performed a comparative analysis in the entire population. The corresponding results showed that patients with LMBC and brain metastasis had the worst survival, and the additional involvement of the bone did not decrease the overall prognosis. Although the metastatic patterns and prognostic correlations have been discussed in previous studies^12,20,21^, they tended to focus on the entire group of patients with de novo metastatic breast cancer instead of patients with LMBC. Therefore, the findings might not apply to patients with newly diagnosed lung involvement. In the current study, we conducted analyses in this specific cohort and reported novel findings of prognostic profiles associated with involved patterns, which can provide promising evidence for clinical management of patients with LMBC in clinical practice.

Given the controversial role of surgical intervention in de novo metastatic breast cancer^22–25^, we comprehensively discussed the potential effects of surgical performance on the prognosis of LMBC. Surgical performance could prolong the OS of patients with LMBC independent of the molecular subtypes. For patients with LMBC with lung-only and paired metastases, this kind of survival benefit remained consistent. Collectively, resection of primary disease can improve the overall prognosis of patients with. LMBC and this benefit tended to vary with metastatic patterns, which was consistent with previous findings^26^. There is a promising rationale for this practice, and increasing evidence has emerged for surgical performance in de novo stage IV breast cancer^27^. However, we could not further elaborate on the correlations between surgical performance and involved patterns in specific breast cancer subtypes due to the limited sample size, in addition to the specific techniques regarding surgery including surgical procedures, the optimal time point for surgery, and predictive biomarkers of the advantageous population for the receipt of surgical intervention due to limited data in the database. In addition, the overall prognosis could be interpreted by a show of factors associated with cancer treatment and disease characteristics in the setting of therapeutic phrases, these findings should be used with enough caution for physicians. However, considering the limited evidence for the prognostic value of surgical intervention for patients with LMBC, the current study could provide emerging evidence, and further studies should be conducted to investigate the associations between primary disease resection and surgical performance in the specified cohorts from the LMBC population.

To further quantify the estimation for individual prognosis, we developed a prediction model for the 2- and 5-year survival probabilities of patients with. de novo LMBC, which was further validated internally and externally in the selected cohorts. The results of model validation suggested that this novel nomogram provided a robust prediction of survival in the LMBC population. Considering that this reliable nomogram was the first fulfillment of prognostic estimation for LMBC, the present study provides strong evidence for practitioners to introduce individual-based therapeutics for survival benefits in clinical practice.

There are limitations to our findings. First, metastatic sites were not fully recorded in this database, which comprised the metastatic sites after sequential therapies and the soft tissue and distant lymph nodes at the initial diagnosis, and could exert inevitable effects on the proportion of results regarding metastatic patterns. However, the organs commonly involved in breast cancer include the lung, bone, liver, and brain^28^, which were included in our analyses, and the study results can be applied to all patients with LMBC. In addition, treatment information was not sufficiently available. This includes, for instance, endocrine therapy as a first-line intervention for ER+/HER2− breast cancer, targeted therapy for HER2+ breast cancer, chemotherapeutic protocols, radiation performance, and surgical removal of metastatic lesions, which could result in misestimation of the associations between current treatment options and survival benefits as well as ignorance of the influence of some new treatments, such as immunotherapy, PARP inhibitors, and PI3K-AKT inhibitors on survival benefits. This should be further improved in future population-based studies. Moreover, information on progression-free survival was not included in the SEER database, leading to a lack of a major survival profile. Finally, several disease characteristics vital to clinical outcomes are absent in this database, such as the Ki-67 index and lymphovascular invasion; therefore, we could consider all disease characteristics to further calibrate this prediction model.

In conclusion, this study revealed great heterogeneity in the clinical outcomes of LMBC associated with molecular subtypes, metastatic patterns, and surgical performance. Prognostic factors were identified, and we established a robust nomogram for the estimation of individual 2- and 5-year survival in patients with LMBC. Prospective studies with more cohorts for extensive validation are warranted in the future.

## Supplementary Information

Supplementary Information.

## Data Availability

The SEER database was available from: www.seer.cancer.gov.
