# Supplementary material for: Clinical outcomes and a nomogram for de novo metastatic breast cancer with lung metastasis: a population-based study
Source: Sci Rep. 2022 Mar 4;12:3597. doi: 10.1038/s41598-022-07565-x (PMC8897413; doi:10.1038/s41598-022-07565-x)
Supplement: Supplementary file 1 — Supplementary Information. [file 41598_2022_7565_MOESM1_ESM.docx]

Supplementary Materials

Supplementary Table 1. Population demographics and baseline characteristics of eligible cohort.

| Characteristics | LMBC (N=4310) | |
| --- | --- | --- |
|  | No. | Percent (%) |
| Age, y | 62.0 |  |
| Age group, y |  |  |
| <50 | 759 | 17.6 |
| 50-69 | 2233 | 51.8 |
| ≥70 | 1318 | 30.6 |
| Sex |  |  |
| Female | 4245 | 98.5 |
| Male | 65 | 1.5 |
| Race |  |  |
| White | 3096 | 71.8 |
| Black | 817 | 19.0 |
| Others | 397 | 9.2 |
| Laterality |  |  |
| Left | 2174 | 50.4 |
| Right | 2114 | 49.0 |
| Others | 22 | 0.5 |
| Histologic type |  |  |
| DC | 3565 | 82.7 |
| LC | 138 | 3.2 |
| Others | 607 | 14.1 |
| Grade |  |  |
| Grade1 | 241 | 5.6 |
| Grade2 | 1627 | 37.7 |
| Grade3 | 2403 | 55.8 |
| Grade4 | 39 | 0.9 |
| Subtype |  |  |
| HR+/HER2- | 2259 | 52.4 |
| HR+/HER2+ | 757 | 17.6 |
| HER2 | 467 | 10.8 |
| TN | 827 | 19.2 |
| ER |  |  |
| Positive | 2932 | 68.0 |
| Negative | 1378 | 32.0 |
| PR |  |  |
| Positive | 2389 | 55.4 |
| Negative | 1921 | 44.6 |
| HER2 |  |  |
| Positive | 1224 | 28.4 |
| Negative | 3086 | 71.6 |
| T |  |  |
| T0 | 12 | 0.3 |
| T1 | 463 | 10.7 |
| T2 | 1224 | 28.4 |
| T3 | 761 | 17.7 |
| T4 | 1850 | 42.9 |
| N |  |  |
| N0/N1mi | 1033 | 24.0 |
| N1 | 2062 | 47.8 |
| N2 | 556 | 12.9 |
| N3 | 659 | 15.3 |
| Bone involvement |  |  |
| Yes | 2280 | 52.9 |
| No | 2030 | 47.1 |
| Liver involvement |  |  |
| Yes | 1182 | 27.4 |
| No | 3128 | 72.6 |
| Brain involvement |  |  |
| Yes | 422 | 9.8 |
| No | 3888 | 90.2 |
| Surgery |  |  |
| Yes | 1285 | 29.8 |
| No/Unknown | 3025 | 70.2 |
| Radiotherapy |  |  |
| Yes | 1234 | 28.6 |
| No/Unknown | 3076 | 71.4 |
| Chemotherapy |  |  |
| Yes | 2619 | 60.8 |
| No/Unknown | 1691 | 39.2 |

Supplementary Table 2. The incidence of metastatic patterns of included LMBC patients.

| Characteristics | LMBC (N=4310) |
| --- | --- |
|  | No. (%) |
| One site |  |
| Lung | 1555 (36.1) |
| Two sites |  |
| Lung and Bone | 1332 (30.9) |
| Lung and Liver | 342 (7.9) |
| Lung and Brain | 97 (2.3) |
| Three sites |  |
| Lung, Bone, and Liver | 658 (15.3) |
| Lung, Bone, and Brain | 144 (3.3) |
| Lung, Liver, and Brain | 36 (0.8) |
| Four sites |  |
| Lung, Bone, Liver, and Brain | 146 (3.4) |

Supplementary Table 3. Comparative analysis of survival outcomes associated with metastatic patterns.

| Lung 30.0 (27.2-32.8) |  |  |  |  |  |  |  | | |
| --- | --- | --- | --- | --- | --- | --- | --- | --- | --- |
| 0.053 | Lung and Bone 29.0 (26.6-31.4) |  |  |  |  |  |  | | |
| <0.0001 | <0.0001 | Lung and Liver 14.0 (11.4-16.6) |  |  |  |  |  | | |
| <0.0001 | <0.0001 | 0.002 | Lung and Brain 8.0 (6.1-9.9) |  |  |  |  | | |
| <0.0001 | <0.0001 | 0.621 | 0.002 | Lung, Bone, and Liver 14.0 (11.7-16.3) |  |  |  | | |
| <0.0001 | <0.0001 | 0.737 | 0.004 | 0.457 | Lung, Bone, and Brain 14.0 (7.7-20.3) |  |  | |  |
| <0.0001 | <0.0001 | 0.066 | 0.819 | 0.073 | 0.067 | Lung, Liver, and Brain 10.0 (5.0-14.9) |  |  |  |
| <0.0001 | <0.0001 | <0.0001 | 0.559 | <0.0001 | <0.0001 | 0.468 | Lung, Bone, Liver, and Brain 6.0 (3.3-8.7) |  |  |

Supplementary Table 4. Comparative analysis of survival outcomes stratified by clinical features.

| Characteristics | OS | | *P* value |
| --- | --- | --- | --- |
|  | Mean (95%CI) | Median (95%CI) |  |
| Age group, y |  |  | <0.0001 |
| <50 | 37.1 (34.5-39.6) | 29.0 (25.9-32.1) |  |
| 50-69 | 33.5 (32.1-34.9) | 25.0 (22.9-27.1) |  |
| ≥70 | 26.4 (24.7-28.2) | 17.0 (15.0-19.0) |  |
| Sex |  |  | 0.209 |
| Female | 32.0 (30.9-33.0) | 23.0 (21.7-24.3) |  |
| Male | 36.9 (28.9-44.7) | 29.0 (14.7-43.3) |  |
| Race |  |  | <0.0001 |
| White | 32.9 (31.7-34.1) | 24.0 (22.4-25.6) |  |
| Black | 26.7 (24.6-28.8) | 18.0 (16.1-19.3) |  |
| Others | 37.1 (33.3-40.8) | 32.0 (25.4-38.6) |  |
| Laterality |  |  | 0.889 |
| Left | 31.9 (30.4-33.3) | 23.0 (21.2-24.8) |  |
| Right | 32.1 (30.7-33.6) | 24.0 (22.1-25.9) |  |
| Others | 29.5 (15.6-43.5) | 10.0 (0.2-33.7) |  |
| Histologic type |  |  | 0.019 |
| DC | 32.6 (31.5-33.8) | 24.0 (22.5-25.5) |  |
| LC | 28.5 (23.7-33.3) | 21.0 (16.1-25.9) |  |
| Others | 29.8 (27.1-32.5) | 18.0 (15.0-21.0) |  |
| Grade |  |  | <0.0001 |
| Grade1 | 41.9 (37.6-46.3) | 41.0 (35.8-46.2) |  |
| Grade2 | 36.9 (35.1-38.7) | 31.0 (28.2-33.8) |  |
| Grade3 | 27.8 (26.5-29.1) | 18.0 (16.7-19.3) |  |
| Grade4 | 27.9 (20.7-35.3) | 19.0 (12.1-25.9) |  |
| Subtype |  |  | <0.0001 |
| HR+/HER2- | 34.6 (33.2-36.1) | 28.0 (26.0-29.9) |  |
| HR+/HER2+ | 41.1 (38.4-43.9) | 35.0 (30.1-39.9) |  |
| HER2 | 32.3 (28.9-35.7) | 22.0 (18.1-25.9) |  |
| TN | 16.7 (15.2-18.1) | 11.0 (10.0-11.9) |  |
| ER |  |  | <0.0001 |
| Positive | 36.7 (35.4-37.9) | 31.0 (28.9-33.0) |  |
| Negative | 22.3 (20.8-23.9) | 13.0 (11.9-14.0) |  |
| PR |  |  | <0.0001 |
| Positive | 37.6 (36.2-39.0) | 32.0 (29.7-34.3) |  |
| Negative | 25.0 (23.6-26.4) | 15.0 (13.8-16.2) |  |
| HER2 |  |  | <0.0001 |
| Positive | 37.8 (35.7-39.9) | 30.0 (26.8-33.2) |  |
| Negative | 29.8 (28.7-31.0) | 20.0 (18.6-21.4) |  |
| T |  |  | <0.0001 |
| T0 | 15.5 (8.5-22.5) | 10.0 (3.8-16.2) |  |
| T1 | 36.7 (33.5-39.9) | 27.0 (23.1-30.9) |  |
| T2 | 34.9 (32.9-36.9) | 28.0 (25.6-30.4) |  |
| T3 | 31.9 (29.5-34.5) | 22.0 (18.8-25.2) |  |
| T4 | 28.8 (27.3-30.3) | 19.0 (17.3-20.7) |  |
| N |  |  | 0.284 |
| N0/N1mi | 31.9 (29.8-34.0) | 22.0 (19.3-24.7) |  |
| N1 | 31.7 (30.1-33.2) | 24.0 (22.2-25.8) |  |
| N2 | 33.8 (31.0-36.5) | 25.0 (20.1-29.9) |  |
| N3 | 31.3 (28.8-33.9) | 22.0 (18.8-25.1) |  |
| Bone involvement |  |  | <0.0001 |
| Yes | 30.2 (28.8-31.5) | 22.0 (20.3-23.7) |  |
| No | 34.1 (32.6-35.7) | 25.0 (22.8-27.2) |  |
| Liver involvement |  |  | <0.0001 |
| Yes | 23.1 (21.3-24.8) | 13.0 (11.5-14.5) |  |
| No | 35.3 (34.1-36.6) | 27.0 (25.3-28.7) |  |
| Brain involvement |  |  | <0.0001 |
| Yes | 19.9 (16.9-22.8) | 9.0 (7.3-10.7) |  |
| No | 33.3 (32.2-34.3) | 25.0 (23.5-26.5) |  |
| Surgery |  |  | <0.0001 |
| Yes | 38.7 (36.9-40.6) | 30.0 (27.5-32.5) |  |
| No/Unknown | 28.7 (27.4-29.9) | 20.0 (18.5-21.5) |  |
| Radiotherapy |  |  | 0.840 |
| Yes | 31.7 (29.9-33.5) | 24.0 (21.7-26.3) |  |
| No/Unknown | 32.2 (30.9-33.5) | 23.0 (21.4-24.6) |  |
| Chemotherapy |  |  | <0.0001 |
| Yes | 35.3 (33.9-36.6) | 27.0 (25.2-28.8) |  |
| No/Unknown | 27.1 (25.5-28.6) | 17.0 (14.9-19.1) |  |

Supplementary Table 5. Comparative analysis of survival outcomes associated with treatment pattern.

| Surgery alone  31.2 (34.8-27.7) |  |  |  |  |  | | |
| --- | --- | --- | --- | --- | --- | --- | --- |
| <0.0001 | Surgery plus CT 40.9 (43.9-38.0) |  |  |  |  | | |
| 0.008 | 0.791 | Surgery plus RT 42.0 (48.8-35.2) |  |  |  | | |
| 0.059 | <0.0001 | 0.019 | CT alone 33.5 (35.7-31.4) |  |  | |  |
| 0.127 | <0.0001 | <0.0001 | <0.0001 | CT plus RT  25.4 (27.9-23.0) |  |  |  |
| <0.0001 | 0.467 | 0.970 | <0.0001 | <0.0001 | Surgery plus CT plus RT 41.7 (45.3-38.4) |  |  |

Abbreviations: CT, Chemotherapy; RT, Radiotherapy.

Supplementary Figure 1. Flowchart of patient selection and cohort enrollment.


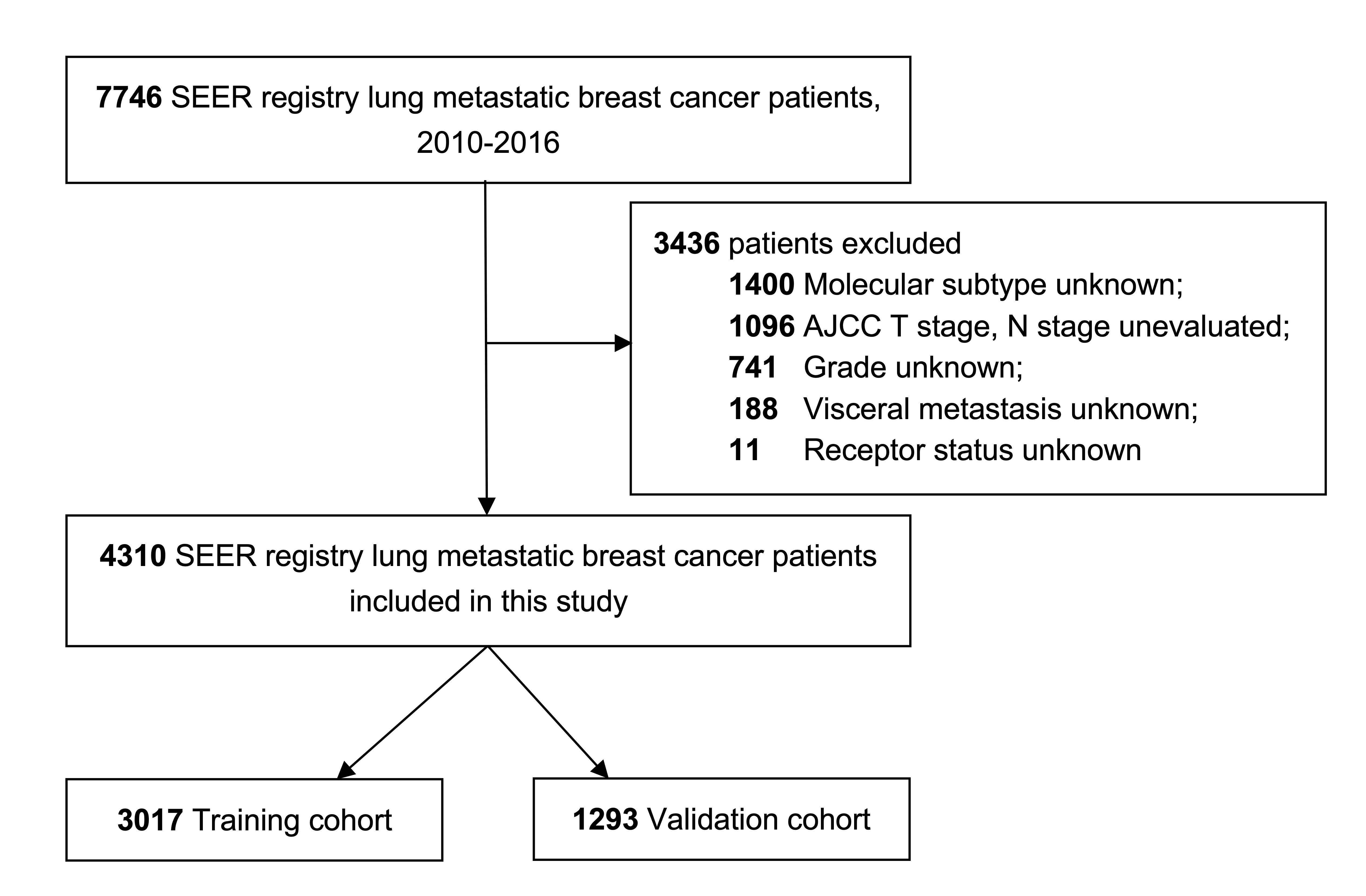


Supplementary Figure 2. Comparative analysis of survival outcomes regarding metastatic patterns.


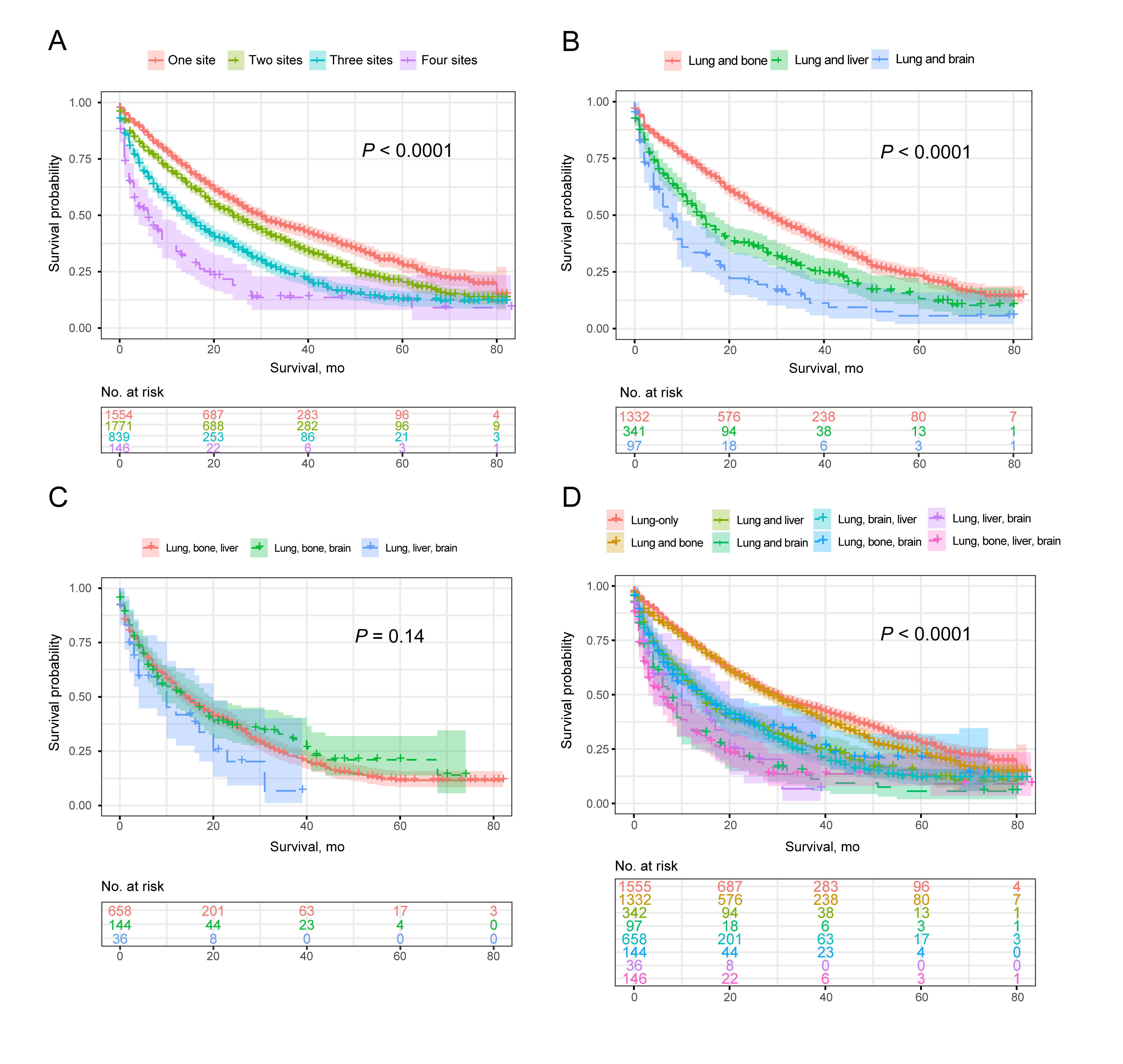


Supplementary
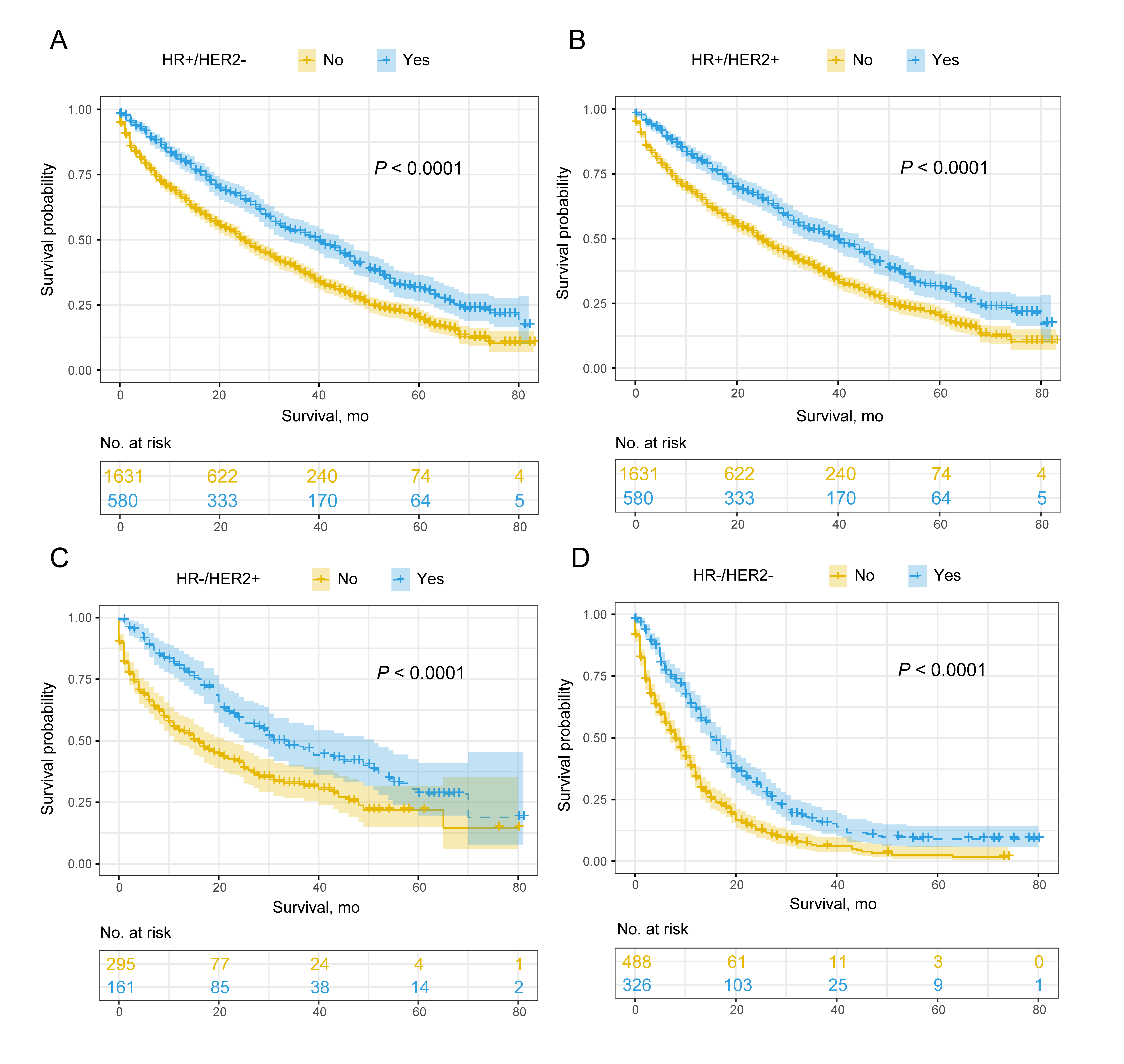
Figure 3. Comparative analysis of survival outcomes associated surgery classified by molecular subtypes.

Supplementary Figure 4. Comparative analysis of survival outcomes associated surgery classified by metastatic patterns.


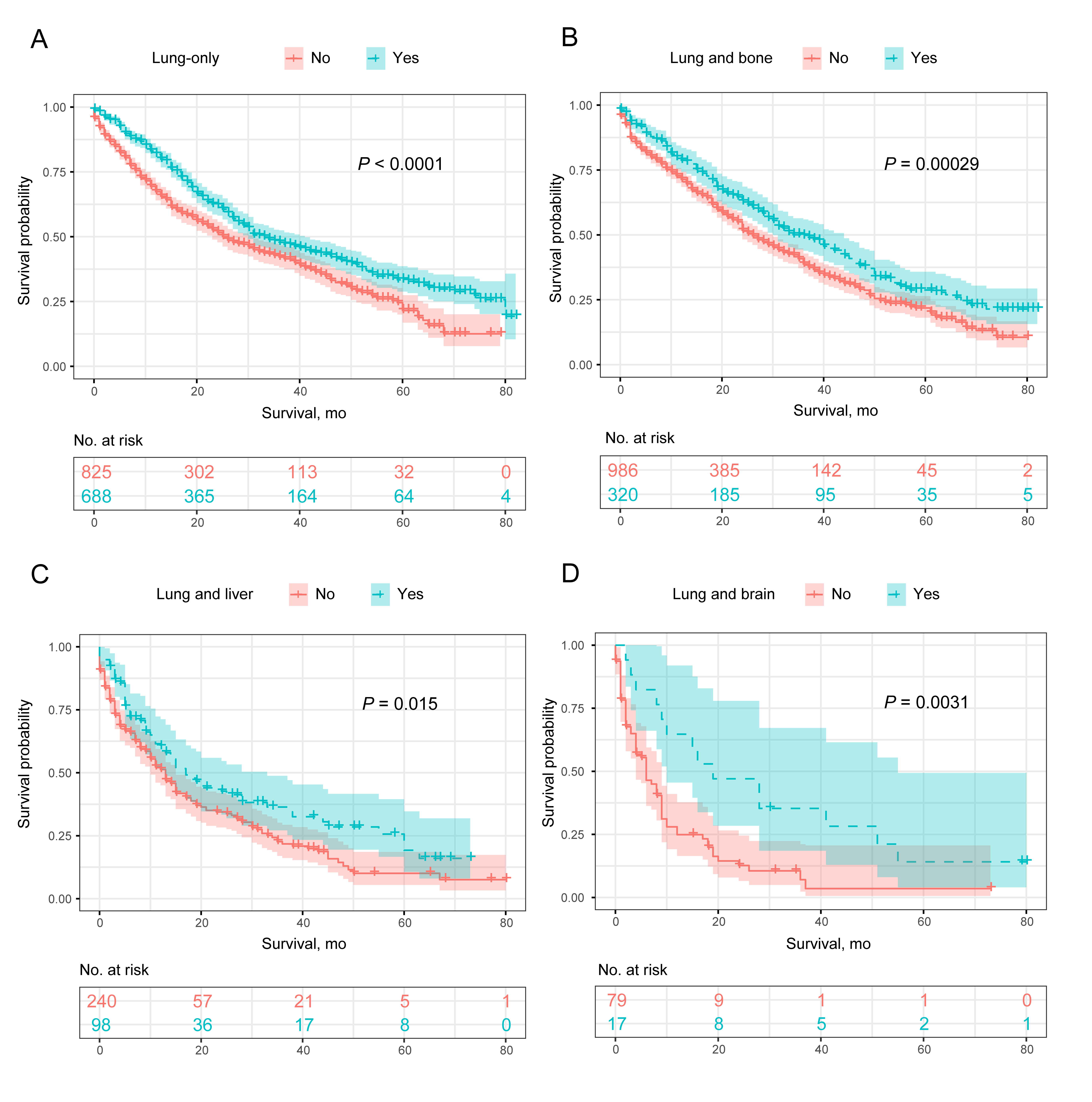


Supplementary Figure 5. Comparative analysis of survival outcomes associated with treatment patterns.


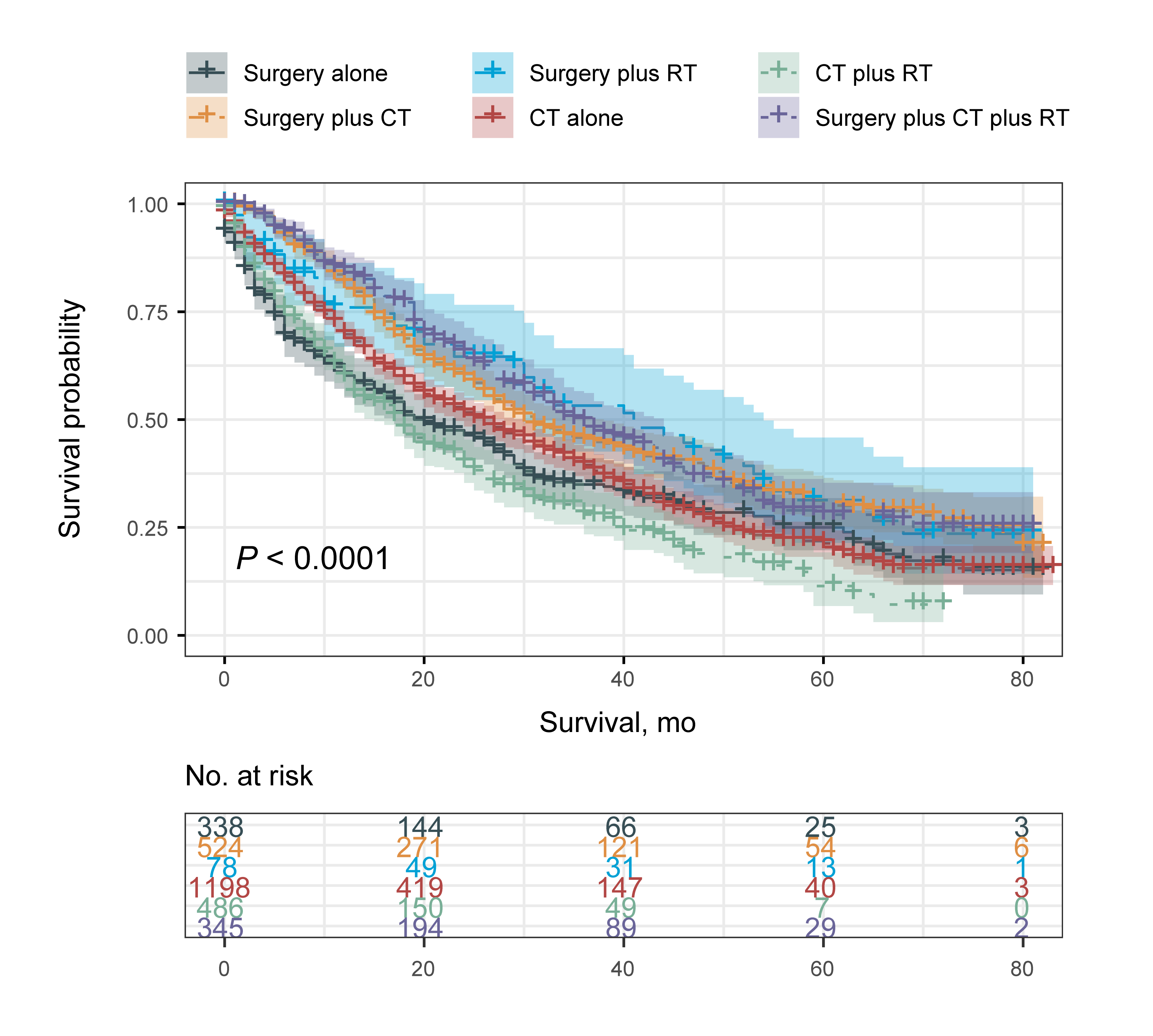


Supplementary Figure 6. Comparative analysis of survival outcomes stratified by risk groups in the training cohort.


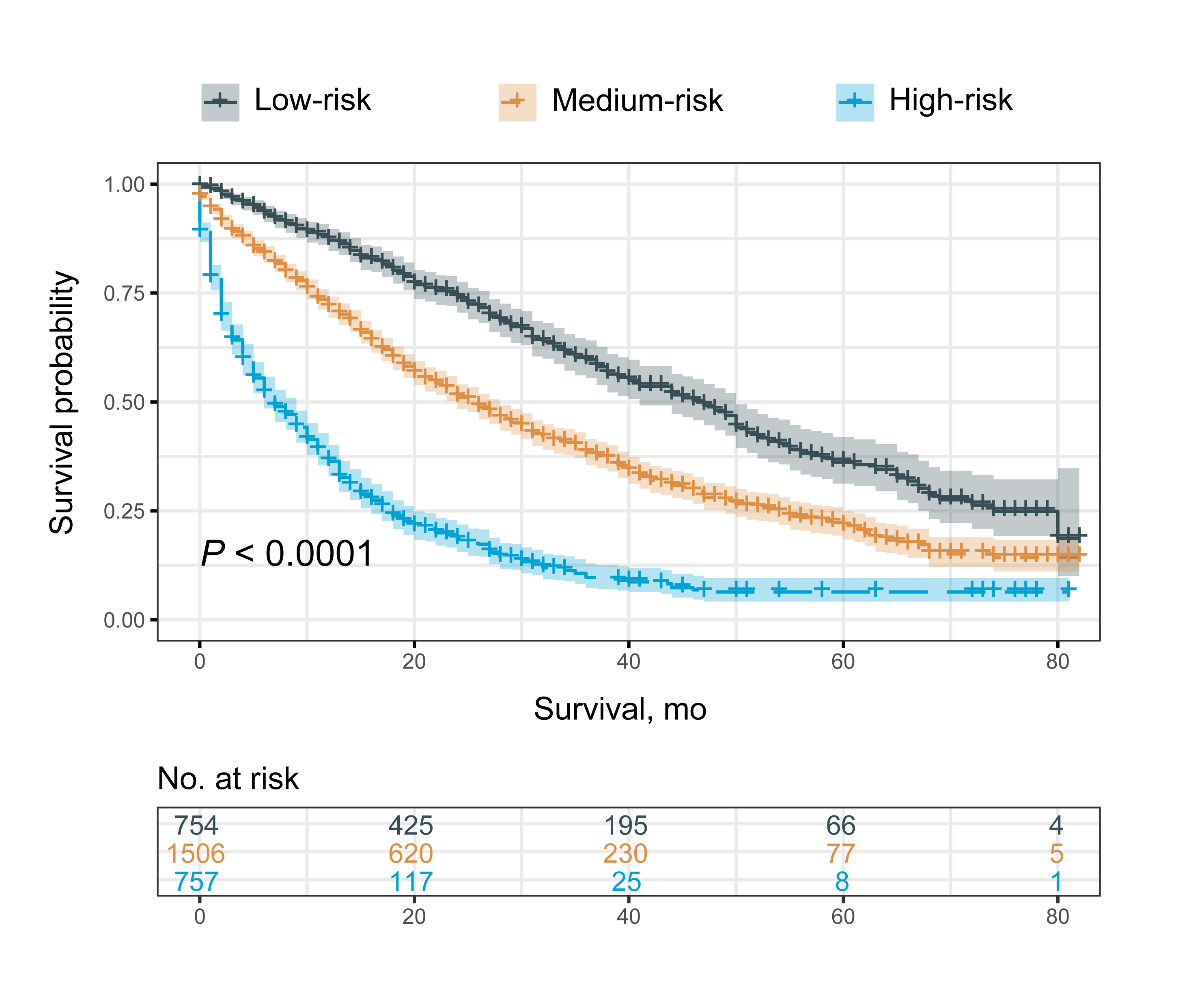


Appendix. Nomogram usage illustration.

For example: 50-year-old black women was diagnosed with lobular, grade III, HR-/HER2+, T4, LMBC tumor with bone-only involvement and the receipt of surgery and chemotherapy would have a total of 31 points and an estimated 2-year of 55% and 5-year OS of 7-8%.

| Variables | Points | Survival Rate | |
| --- | --- | --- | --- |
| 50 years old | 2.9 |  |  |
| Black | 0 |  |  |
| Lobular | 1 |  |  |
| Grade 3 | 3.4 |  |  |
| HR-/HER2+ | 3.3 |  |  |
| T4 | 0 |  |  |
| Bone involvement | 0 |  |  |
| Liver involvement | 5.5 |  |  |
| Brain involvement | 4.3 |  |  |
| Surgery | 5.2 |  |  |
| Chemotherapy | 5.7 |  |  |
| Total points | ≈31 |  |  |
| 2-year survival rate |  | 55% |  |
| 5-year survival rate |  | 7-8% |  |
